# Nephrogenic Diabetes Insipidus: Three Decades of Clinical Reality

**DOI:** 10.7759/cureus.89635

**Published:** 2025-08-08

**Authors:** Mariana Gouveia Lopes, Daniela T Peixoto, Catarina Neves, Marta Machado, Carolina Cordinhã, Carmen Carmo, Clara Gomes

**Affiliations:** 1 Pediatrics, Unidade Local de Saúde da Região de Leiria, Leiria, PRT; 2 Pediatrics, Unidade Local de Saúde da Região de Aveiro, Aveiro, PRT; 3 Pediatric Nephrology, Hospital Pediátrico, Unidade Local de Saúde de Coimbra, Coimbra, PRT

**Keywords:** antidiuretic hormone, genetic study, nephrogenic diabetes insipidus, polydipsia, polyuria

## Abstract

Introduction

Nephrogenic diabetes insipidus (NDI) is a rare condition caused by renal resistance to the action of antidiuretic hormone (ADH) at the level of the distal tubule, resulting in impaired urinary concentration and consequent polyuria. NDI may be hereditary, most commonly X-linked due to AVPR2 gene mutations, or acquired.

Objective

To characterize the clinical features, management strategies, and outcomes of patients with NDI followed at a tertiary pediatric nephrology center.

Methods

A retrospective observational study was conducted, including pediatric patients diagnosed with NDI between 1991 and 2024. Data regarding clinical presentation, diagnostic approach, management strategies, and long-term outcomes were collected and analyzed.

Results

Eight patients (including one set of twins) were identified; seven (87.5%) were male subjects. The median age at diagnosis was 7.5 months (interquartile range (IQR): 6.0-9.0). All patients presented with polydipsia, polyuria (median diuresis: 10.0 (IQR: 9.0-10.2) mL/kg/h), and failure to thrive. Constipation and recurrent fever were observed in two patients (25.0%). At diagnosis, the median serum sodium level was 160.5 mmol/L (IQR: 139-168), and the urine/plasma osmolality ratio was 0.41 (IQR: 0.26-0.49). No patients exhibited metabolic acidosis. All desmopressin tests were negative. Genetic testing was performed in four patients, all of whom had pathogenic X-linked AVPR2 variants.

All patients were treated with hydrochlorothiazide and amiloride; indomethacin was added in 5 (62.5%). Serum sodium normalized in all cases. Over a median follow-up period of 16.9 years (IQR: 8.4-17.4), growth improved, but all patients continued to exhibit polyuria and polydipsia. Neurodevelopmental disorders were identified in six patients, including intellectual disability, autism spectrum disorder, language delay, and learning difficulties. Renal function, serum sodium, and imaging findings remained stable throughout follow-up. At the final assessment, all patients remained on hydrochlorothiazide and amiloride. Five transitioned to adult nephrology at a median age of 17.0 years (IQR: 16.0-17.0).

Conclusion

Although rare, NDI can significantly impact growth and neurodevelopment. The diagnosis should be considered in infants with failure to thrive, especially when accompanied by polyuria and polydipsia. Early recognition and intervention, even if non-specific, may improve long-term outcomes.

## Introduction

Preserving a stable volume and composition of body fluids depends on the precise regulation of water homeostasis, ensuring a continuous balance between intake and loss [1]. This system of fluid homeostasis operates through three interrelated determinants: the thirst reflex, the appropriate synthesis and secretion of antidiuretic hormone (ADH), and the renal tubular response to its action [1-3].

Congenital nephrogenic diabetes insipidus (NDI) is a rare inherited disorder characterized by renal collecting duct resistance to the action of ADH, resulting in an impaired ability to concentrate urine despite normal or elevated plasma levels of the hormone [4-7]. It manifests as the excretion of large volumes of hypotonic urine, often leading to significant hypernatremia and extracellular fluid depletion, potentially life-threatening if not promptly recognized and appropriately managed [1,4].

Approximately 8-14.5% of the glomerular filtrate is reabsorbed within the collecting ducts, a process tightly regulated by ADH and the medullary hyperosmotic gradient. ADH is synthesized in a circadian manner by the magnocellular neurons of the paraventricular and supraoptic nuclei of the hypothalamus and stored in the posterior pituitary gland, from where it is subsequently released into the systemic circulation [3]. Its antidiuretic effect is initiated upon binding to V2 receptors (arginine vasopressin receptor 2, or AVPR2), located on the basolateral membrane of principal cells in the collecting ducts [1,3,8]. This interaction activates an intracellular signaling cascade, culminating in the phosphorylation and apical translocation of aquaporin-2 (AQP2) water channels from intracellular vesicles. This process renders the apical membrane permeable to water. The synthesis and trafficking of AQP2 are, therefore, tightly regulated by ADH stimulation [1,5,9].

When hypothalamic synthesis, pituitary storage, and release of ADH remain intact, the disorder is due to renal tubular insensitivity to the hormone and is pathognomonic of NDI [1,5,8].

In addition to congenital NDI, acquired forms may develop secondary to certain drugs including lithium, amphotericin B, and rifampicin; hematologic malignancies such as leukemia and lymphoma; metabolic disturbances such as hypokalemia and hypercalcemia, as well as medullary injury, chronic pyelonephritis, amyloidosis, chronic kidney disease, urinary tract obstruction, and other less common etiologies [3,5,9].

Hereditary NDI comprises two forms. The predominant form, X-linked NDI, caused by loss-of-function variants in the AVPR2 gene on Xq28, accounts for 90-98% of cases. The remaining 2-10% follow autosomal recessive or dominant inheritance and arise from pathogenic variants in the AQP2 gene on chromosome 12q13.12 [5-7]. To date, over 300 distinct pathogenic AVPR2 loss-of-function variants have been reported in more than 300 families with NDI [5].

NDI predominantly affects male subjects, with heterozygous female subjects usually asymptomatic carriers. However, female subjects may exhibit clinical disease, particularly in the presence of biallelic AQP2 pathogenic variants [5,6]. Nonetheless, some female subjects carrying pathogenic AVPR2 variants (around 10%) may also develop the full NDI phenotype. This phenomenon is likely explained by skewed X-chromosome inactivation, favoring the X chromosome that does not carry the pathogenic AVPR2 variant [6].

In early 2025, an international consortium of leading endocrinology societies recommended renaming diabetes insipidus to ADH deficiency, and NDI to ADH resistance [10]. The primary rationale for this proposed change was to clearly distinguish these conditions from diabetes mellitus [10]. However, this new terminology has not yet been officially adopted and is not currently included in the International Statistical Classification of Diseases and Related Health Problems [6].

This study aims to retrospectively characterize the clinical features, biochemical profile, treatment approaches, and long-term outcomes of children diagnosed with nephrogenic diabetes insipidus, followed in a tertiary pediatric nephrology center over a 34-year period.

## Materials and methods

We conducted a descriptive observational study with retrospective data collection, including all children diagnosed with nephrogenic diabetes insipidus, followed in the Pediatric Nephrology Unit of a tertiary care hospital, over a 34-year period, between January 1991 and December 2024.

The following parameters were evaluated: sex, age at diagnosis, duration of follow-up, clinical presentation, diagnostic investigations, treatment, and clinical course. Initial diuresis was assessed over a 24-hour period during inpatient admission. Polyuria was defined as a persistent urine output > 3 mL/kg/h or > 80 mL/m²/h. Polydipsia was not quantitatively assessed. The diagnostic evaluation included serum urea, creatinine, sodium, blood gas analysis, and plasma osmolality [osm(p)], alongside urinary specific gravity and osmolality [osm(u)]. Hypernatremia was defined as serum sodium > 145 mmol/L; plasma hyperosmolality as osm(p) > 295 mOsm/kg; urinary hypo-osmolality as osm(u) < 250 mOsm/kg; and low urinary specific gravity as < 1.005. An osm(u)/osm(p) ratio ≥ 1.51 was considered physiologically appropriate.

All patients underwent renal ultrasonography at diagnosis and during follow-up. Targeted genetic testing was conducted when feasible. The water deprivation test, combined with administration of desmopressin (a synthetic analogue of antidiuretic hormone), was employed to confirm the diagnosis. Water restriction was contraindicated in cases with plasma osmolality (osm(p)) > 295 mOsm/kg and/or serum sodium > 145 mmol/L; in such instances, only the desmopressin challenge was performed. Desmopressin was particularly indicated in patients for whom water restriction was contraindicated. A persistently low urinary-to-plasma osmolality ratio (osm(u)/osm(p)) < 1.5 was considered diagnostic of NDI.

Statistical analyses were performed using the IBM SPSS Statistics for Windows, version 29 (IBM Corp., Armonk, NY, USA).

Population characteristics were described using measures of central tendency and dispersion for quantitative variables, and absolute and relative frequencies for qualitative variables.

## Results

A total of eight children with a confirmed diagnosis of NDI were included, seven of whom were male subjects. Two patients were dizygotic twins. A positive family history of NDI was documented in five cases. The median age at diagnosis was 7.5 (interquartile range (IQR): 6.0-9.0) months, and the median follow-up duration was 16.9 (IQR: 8.4-17.4) years.

Clinical presentation

Seven children presented with a typical clinical picture characterized by failure to thrive, accompanied by polydipsia and polyuria (median urine output: 10 (IQR: 9.0-10.2) mL/kg/h). Two children also exhibited constipation and recurrent fever of unknown origin. The median age of onset of failure to thrive (FFT) was 3.5 (IQR: 2.0-4.0) months. Complementary diagnostic tests revealed hypernatremia (median 159 (IQR: 153.0-164.0) mmol/L) and elevated plasma osmolality (median 310 (IQR: 304.0-356.0) mOsm/kg), concomitant with low urine specific gravity (median 1003 (IQR: 1001-1004)) and hypo-osmolality (median 137 (IQR: 80.0-152.0) mOsm/kg). The median urinary-to-plasma osmolarity ratio (osm(u)/osm(p)) was 0.41 (IQR: 0.26-0.49). None of the patients presented with metabolic acidosis.

One patient, diagnosed later at 18 months of age, had exhibited failure to thrive since the age of two months. A family history of cystic fibrosis (two paternal second-degree cousins) prompted the performance of two sweat tests at 6 and 8 months, both of which yielded elevated results (235 and 272 mOsm/kg). Despite the absence of classical clinical features, such as recurrent respiratory infections or chronic diarrhea, a presumptive diagnosis of cystic fibrosis was initially made. At 18 months, during an episode of gastroenteritis, laboratory investigations revealed marked hypernatremia (167 mmol/L), elevated urea (20 mmol/L) and creatinine (80 μmol/L), along with low urine specific gravity (1005). During hospitalization, polyuria and polydipsia were observed, raising suspicion for NDI, which was subsequently confirmed by a lack of response to desmopressin (Table 1).

**Table 1:** Clinical characteristics and complementary investigations FTT: failure to thrive; (u): urinary; (p): plasmatic ^a^dizygotic twins

Case	A ♂	B ♂	C ♂	D^a ^♂	E^a ^♂	F ♀	G ♂	H ♂
Age at diagnosis (months)	7	6	18	9	9	8	2	6
Age at onset of FTT (months)	3	4	2	4	4	2	2	2
Urine output (mL/kg/h)	10	9	6.6	9	12	10.2	10	-
Serum sodium (mmol/L)	159	153	168	164	166	139	162	156
Specific gravity (u)	1005	1003	1001	1003	1005	1001	1002	1004
Osmolarity u (mOsm/kg)	52	80	-	137	152	116	149	172
Osmolarity p (mOsm/kg)	305	310	347	356	359	285	304	315
Osmolarity ratio (u/p)	0.38	0.45	0.17	0.26	0.4	0.41	0.49	0.51

Diagnosis

All children underwent a desmopressin challenge test, which yielded negative results in every case. Renal and bladder ultrasonography was performed in all children and revealed no structural abnormalities.

Genetic analysis was performed in four patients and identified pathogenic variants in the AVPR2 gene, confirming X-linked inheritance.

Treatment

All children were initiated on a low-sodium diet with tailored fluid intake. Pharmacological therapy included hydrochlorothiazide (2 mg/kg/day) and amiloride (0.3 mg/kg/day). Indomethacin was introduced in five patients between 1.5 and 5 months after diagnosis. Oral potassium chloride supplementation was required in three children.

Clinical course

During follow-up, all patients exhibited catch-up growth and continued along normal growth trajectories, despite ongoing polyuria and polydipsia. Neurodevelopmental disorders were identified in six children: five were diagnosed with specific learning difficulties, including two dizygotic twins who also met criteria for intellectual developmental disorder and autism spectrum disorder, while one child presented with an expressive language delay.

All patients achieved normal serum sodium levels, and renal function and renal/bladder ultrasound findings remained normal throughout follow-up.

Currently, three children remain under pediatric nephrology follow-up, with a median age of six years (IQR: 3-6), all continuing treatment with hydrochlorothiazide and amiloride. The remaining five children have transitioned to adult nephrology care, with a median age of 17 years (IQR: 16.0-17.0). All maintain normal renal function and serum sodium levels and continue treatment with hydrochlorothiazide and amiloride, with indomethacin added in four cases (Table 2).

**Table 2:** Clinical course ASD: autism spectrum disorder; IDD: intellectual developmental disorder; HCTZ: hydrochlorothiazide; IND: indomethacin ^a^dizygotic twins

Case	A ♂	B ♂	C ♂	D^a^ ♂	E^a^ ♂	F ♀	G ♂	H ♂
Age at last follow-up (months)	15	15	17	17	17	12	6	3
Clinical course	Catch-up growth; Improvement in polyuria/polydipsia; Normalization of serum sodium levels; Preserved renal function							
Neurodevelopment	Specific learning disabilities	Specific learning disabilities	Specific learning disabilities	Specific learning disabilities	Specific learning disabilities	-	-	Expressive language delay
	-	-	-	ASD/IDD	ASD/IDD	-	-	-
Therapeutic regimen	HCTZ+ amiloride	HCTZ+ amiloride	HCTZ+ Amiloride	HCTZ+ amiloride	HCTZ+ amiloride	HCTZ+ amiloride	HCTZ+ amiloride	HCTZ+ amiloride
	IND	IND	IND	IND	IND	-	-	-
	Low-sodium diet	Low-sodium diet	Low-sodium diet	Low-sodium diet	Low-sodium diet	Low-sodium diet	Low-sodium diet	Low-sodium diet

## Discussion

Only eight cases of NDI were diagnosed over a period exceeding 30 years, underscoring the rarity of this condition, despite it being the most common cause of ADH resistance in children [5,9].

The hallmark clinical features of NDI include polyuria, which may lead to dehydration, and secondary polydipsia. These symptoms are frequently overlooked in early infancy and are often only recognized retrospectively during detailed history-taking [8,9]. Infants with NDI typically present with a heterogeneous spectrum of symptoms, such as feeding difficulties, vomiting, recurrent unexplained fever, failure to thrive, constipation, lethargy, and irritability, often attributable to restricted fluid intake [5,6]. Hypernatremia and hyperchloremia are common biochemical findings [10], whereas nocturia and enuresis tend to emerge later in childhood [5,11].

In our series, all infants presented with poor weight gain in association with polyuria and polydipsia, and some also exhibited constipation and recurrent febrile episodes. The median age at diagnosis was nine months, in line with existing literature indicating that most patients are diagnosed within the first year of life [5,7]. In one case, polyuria and polydipsia were not initially apparent, and diagnosis was delayed due to two positive sweat tests, which led to a preliminary suspicion of cystic fibrosis.

Patients with NDI are typically born at term [6]. In utero polyuria is often mild or absent, and polyhydramnios is generally not observed. Clinical manifestations may emerge within the first weeks of life but are often subtle, particularly in exclusively breastfed infants [6,9].

Human breast milk, being relatively low in salt and protein, imposes a limited osmotic load, thereby reducing urinary output and mitigating the risk of hypernatremia. In contrast, formula feeding and the introduction of solid foods increase renal solute load, exacerbating polyuria. As a result, exclusively breastfed infants may experience a delayed diagnosis [6,9]. In our study, none of the infants were exclusively breastfed at the time of symptom onset.

Initial assessment should include measurement of urine and plasma osmolality, as well as serum sodium concentration. The presence of inappropriately dilute urine in the context of elevated or high-normal serum sodium is pathognomonic for either central or nephrogenic diabetes insipidus [1,5,6].

According to Levtchenko et al., the current clinical practice guidelines from the European Society for Paediatric Nephrology recommend considering a diagnosis of NDI in infants and children presenting with polyuria, polydipsia, failure to thrive, and hypernatremic dehydration [6].

All children in our series underwent a desmopressin challenge test, with uniformly negative results. This lack of response is consistent with polyuria of renal origin, as seen in NDI [9].

Water deprivation testing is contraindicated in the presence of hypernatremia (serum sodium > 145 mmol/L) due to the risk of severe dehydration. In patients with elevated or high-normal serum sodium and inappropriately dilute urine, the diagnosis of diabetes insipidus can be established without the need for water restriction [5,6,11].

Genetic analysis was performed in four children and identified pathogenic variants in the AVPR2 gene, consistent with X-linked inheritance [5,6]. Notably, one of these patients was a female subject, an uncommon finding, as female subjects are typically asymptomatic carriers. However, in this case, the patient exhibited overt clinical features of NDI. Genetic testing can provide a definitive diagnosis of NDI and has important implications for clinical prognosis and genetic counselling. Early genetic testing is particularly recommended in at-risk children, such as male offspring of known carrier mothers [5,6,12]. In families without a prior history of NDI, de novo pathogenic variants may be identified, facilitating risk assessment and counselling for extended family members [5,6].

Currently, there is no curative treatment for NDI. Management focuses on reducing polyuria, preventing dehydration and hypernatremia, and minimizing long-term complications [9]. Treatment comprises both non-pharmacological and pharmacological strategies.

Non-pharmacological measures include maintaining adequate hydration and implementing a low-sodium, low-protein diet to reduce osmotic diuresis [5,6,11]. During infancy, a low renal solute load, approximately 15 mOsm/kg/day, is recommended to minimize polyuria and polydipsia, improve feeding tolerance, and support growth [5,6].

Young children require frequent water intake, including overnight, to prevent dehydration and hypernatremia. A low-sodium, nutritionally adequate diet is essential. For infants, age-appropriate milk intake must be maintained to ensure sufficient caloric intake [5,6,11]. Nasogastric feeding is advised in cases of persistent vomiting, recurrent dehydration, or failure to thrive, as it ensures adequate fluid, energy, and nutrient intake [5-7].

If symptoms persist despite non-pharmacological interventions, initiation of therapy with a thiazide diuretic is recommended. Thiazides exert their effect by inducing mild volume depletion and, when combined with a low-sodium diet, can acutely reduce urine output by up to 50% [1,2,5,6,11]. This effect is presumably mediated by hypovolemia-induced enhancement of proximal tubular sodium and water reabsorption, thereby decreasing the volume of fluid delivered to the collecting ducts and subsequently reducing diuresis [5,6]. However, although thiazide diuretics have demonstrated favorable outcomes in the early phases of treatment, their efficacy tends to decline over time [6,7,11]. Long-term use of hydrochlorothiazide may be associated with adverse effects such as hypokalemia and cardiac arrhythmias. The addition of amiloride, a potassium-sparing diuretic, has demonstrated improved efficacy and a more favorable side-effect profile [2,5-7,11].

Hydrochlorothiazide and indomethacin may also be used in combination, particularly during infancy when clinical management is more challenging [5,6,11]. These agents can have pronounced initial effects, including the risk of seizures due to rapid correction of serum sodium levels [6,7]. Indomethacin is additionally associated with potential renal, gastrointestinal, and hematological adverse effects [5,6]. In our study, due to a suboptimal initial response to the combination of hydrochlorothiazide and amiloride, indomethacin was added to the treatment regimen, resulting in a favorable clinical response without the occurrence of the commonly reported side effects [5,6,11]. Close clinical and biochemical monitoring is essential to mitigate and promptly identify potential complications [5,6].

NDI is a rare disorder with unknown true incidence and prevalence, and limited long-term data regarding clinical outcomes and complications [6,7]. As with many inherited conditions, NDI exhibits a wide spectrum of clinical severity. The most serious consequences occur in patients who receive inadequate treatment, often experiencing recurrent episodes of hypernatremic dehydration, which may result in brain injury and long-term cognitive impairment [7,12,13]. Studies specifically investigating this association have reported the presence of cognitive impairment in patients who also fulfill diagnostic criteria for attention-deficit/hyperactivity disorder. The prevalence of these comorbidities varies among studies but is consistently higher than that observed in the general population [13-15]. However, cognitive development is expected to be normal if the diagnosis is made early and episodes of dehydration are adequately prevented [5]. In our study, six children developed neurodevelopmental disorders: five had specific learning difficulties, two of whom (dizygotic twins) had a diagnosis of autism spectrum disorder and intellectual developmental disorder, and one child had expressive language delay. Some studies have suggested a potential link between vasopressin receptor polymorphisms and autism spectrum disorder. However, these associations involve AVPR1 gene polymorphisms, not AVPR2 [16].

Other long-term complications described in the literature include short stature; however, all children in our cohort demonstrated catch-up growth during follow-up. Hydronephrosis, bladder dysfunction, and enuresis have also been reported [7,13,14], although none of these complications were observed in our study. Previous studies have shown that the prevalence of chronic kidney disease is significantly higher in patients with NDI compared to the general population, with impaired renal function being associated with an increased risk of urological complications [5]. In our cohort, serum sodium levels normalized, and both renal function and renal ultrasonography findings remained within normal limits throughout the follow-up period.

This study has several limitations that warrant consideration. Firstly, the small sample size (eight patients) reflects the rarity of NDI, but limits the statistical power of the study and the generalizability of the findings. Secondly, the retrospective nature of the study may introduce bias due to the reliance on medical records, which are subject to inconsistencies or incomplete documentation, particularly in earlier decades of follow-up.

## Conclusions

Congenital NDI is a rare but critical diagnosis to consider in infants presenting with failure to thrive. Polyuria and polydipsia may not be readily apparent in early childhood, which can delay recognition. Management remains challenging due to the absence of curative therapy; however, early initiation of treatment can substantially improve clinical outcomes. A detailed family history and genetic testing are fundamental for establishing the diagnosis and for identifying additional affected individuals. Ongoing clinical and laboratory monitoring is essential to prevent disease-related complications and to optimize long-term prognosis.
